# WGCNA and molecular docking identify hub genes for cardiac aging

**DOI:** 10.3389/fcvm.2023.1146225

**Published:** 2023-04-27

**Authors:** Ping Ping, Lixun Guan, Chaoxue Ning, Qiong Liu, Yali Zhao, Xiang Zhu, Ting Yang, Shihui Fu

**Affiliations:** ^1^ General Station for Drug and Instrument Supervision and Control, Joint Logistic Support Force of Chinese People's Liberation Army, Beijing, China; ^2^ Hematology Department, Hainan Hospital of Chinese People's Liberation Army General Hospital, Sanya, China; ^3^ Central Laboratory, Hainan Hospital of Chinese People's Liberation Army General Hospital, Sanya, China; ^4^ Medical Care Center, Hainan Hospital of Chinese People's Liberation Army General Hospital, Sanya, China; ^5^ Department of Infectious Disease, Army No.82 Group Military Hospital, Baoding, China; ^6^ Department of Cardiology, Hainan Hospital of Chinese People's Liberation Army General Hospital, Sanya, China; ^7^ Department of Geriatric Cardiology, Chinese People's Liberation Army General Hospital, Beijing, China

**Keywords:** cardiac aging, bioinformatics, GEO, immunity, anti-aging

## Abstract

**Background:**

Cardiac aging and ageing-related cardiovascular diseases remain increase medical and social burden. Discovering the molecular mechanisms associated with cardiac aging is expected to provide new perspectives for delaying aging and related disease treatment.

**Methods:**

The samples in GEO database were divided into older group and younger group based on age. Age-associated differentially expressed genes (DEGs) were identified by limma package. Gene modules significantly associated with age were mined using weighted gene co-expression network analysis (WGCNA). Protein-protein interaction networks (PPI) networks were developed using genes within modules, and topological analysis on the networks was performed to identify hub genes in cardiac aging. Pearson correlation was used to analyze the association among hub genes and immune and immune-related pathways. Molecular docking of hub genes and the anti-aging drug Sirolimus was performed to explore the potential role of hub genes in treating cardiac aging.

**Results:**

We found a generally negative correlation between age and immunity, with a significant negative correlation between age and b_cell_receptor_signaling_pathway, fc_gamma_r_mediated_phagocytosis, chemokine signaling pathway, t-cell receptor signaling pathway, toll_like_receptor_signaling_pathway, and jak_stat_signaling_pathway, respectively. Finally, 10 cardiac aging-related hub genes including LCP2, PTPRC, RAC2, CD48, CD68, CCR2, CCL2, IL10, CCL5 and IGF1 were identified. 10-hub genes were closely associated with age and immune-related pathways. There was a strong binding interaction between Sirolimus-CCR2. CCR2 may be a key target for Sirolimus in the treatment of cardiac aging.

**Conclusion:**

The 10 hub genes may be potential therapeutic targets for cardiac aging, and our study provided new ideas for the treatment of cardiac aging.

## 1. Introduction

Epidemiological studies have found that by 2050, the number of people aged 65 and older will exceed 1.5 billion worldwide, accounting for 16% of the global population (1). Cardiac aging and related diseases will increase burden society. Cardiac aging leads to heart failure, and furthermore, aging leads to a declined ability of cardiomyocytes and non-cardiomyocytes to replicate DNA, which in turn causes dysregulation of cellular life processes (2). Cardiac aging can cause cardiovascular diseases such as atrial fibrillation, heart failure and hypertension. The prevalence of these cardiac aging-related diseases is increasing annually (3, 4). The mechanisms of cardiomyocyte senescence mainly include oxidative stress, autophagy and apoptosis (5). However, the precise molecular mechanisms of cardiac aging are not yet clarified. Therefore, in-depth studies are needed to explore the mechanisms for improving the quality of life of patients.

As biotechnology progresses in recent years, research represented by transcriptomics has provided new insight into disease pathogenesis. Bioinformatics has demonstrated significant potential in detecting biomarkers related to disease pathogenesis and progression (6, 7). Currently, there are studies using bioinformatics methods to identify hub genes in the pathogenesis of cardiovascular diseases and heart failure. For instance, Zhao et al. (8) developed a machine learning algorithm-based model for early assessment of heart failure with preserved ejection fraction (HFpEF), which is expected to provide guidance for clinical decision making. Qu et al. (9) identified FAM171B as a novel biomarker of pulmonary arterial hypertension (PAH) using bioinformatics such as WGCNA and SVM, and showed that PAH may be closely associated with FAM171B. Liu et al. (10) pointed out CALU and PALLD as potential biomarkers associated with immune infiltration in heart failure due to ischemic cardiomyopathy. From these studies, it is obvious that bioinformatics tools are playing an essential role in the era of precision medicine. Explosive advances in next-generation sequencers (NGS) and computational analysis to handle large amounts of data have enabled us to comprehensively analyze cancer genome profiles at the research and clinical levels and opening up the possibility of precision medicine, such as RNA sequencing (RNA-seq) (11). Current studies have focused on cardiovascular-related diseases caused by heart failure, and there are no studies directly targeting different age groups to explore the underlying mechanisms of Cardiac aging.

By regulating oxidative stress, inflammation and organelle function, rapamycin (also known by the trade names of sirolimus or rapamune) may inhibit cardiac ageing (12). Studies have shown that rapamycin can reduce mitochondrial reactive oxygen species and inhibit cardiac hypertrophy and cardiac ageing through the inhibition of mTORC1 (13, 14). Rapamycin can directly inhibit mTORC1 and is the first and only macrolide drug approved by the US Food and Drug Administration (FDA).

In this study, we identified age-related co-expressed gene modules by weighted gene co-expression network analysis (WGCNA) through analyzing different age cohort samples in Gene Expression Omnibus (GEO) database, and further identified closely related pathways and hub genes through linking genes in the modules to immune status. Further, molecular docking simulations were performed between anti-aging drugs and hub genes to evaluate pathogenesis and therapeutic targets. In particular, the pathogenesis of cardiac aging were comprehensively investigated to provide so as to new evidence for subsequent in-depth studies.

## 2. Materials and methods

### 2.1. Data acquisition and preprocessing

The RNA-Seq data (FPKM standardized data) and clinical information of chip data sets GSE57338 (136 samples), GSE141910 (166 samples) and GSE173608 (20 samples) as well as the annotation information of chip probes of corresponding platforms were obtained from GEO (https://www.ncbi.nlm.nih.gov/geo/) database. Each data set was processed as follows: (1) Disease samples were removed, and only normal and healthy samples were retained; (2) Samples ≥65 were defined as the elderly, and samples <65 were defined as the young group; (3) Samples with age information and expression value were retained; (4) The expression of samples was transformed into a probe symbol.

### 2.2. Association between age and immunity

We employed the estimation of stromal and Immune cells in malignant tumour tissues using expression data (ESTIMATE) algorithm (15) to calculate the StromalScore, ImmuneScore, and ESTIMATEScore for samples in the GSE57338 cohort. Next, the Pearson correlation between age and the three scores was respectively calculated, and the differences in the distribution of the three scores between the older and younger groups were examined. Finally, we employed the method of single sample gene set enrichment analysis (ssGSEA) (16) to assess pathway enrichment scores in c2.cp.kegg.v7.4.symbols.gmt and screened for biological pathways significantly associated with age by Pearson correlation (*p *< 0.05).

### 2.3. Identification of differentially expressed genes (DEGs) for cardiac aging

We carried out a differential expression analysis between the two groups of samples in the GSE57338 cohort using the limma package (17) under the threshold criterion for DEGs set at |logFoldChang (FC) >1.2 and *p* < 0.05.

### 2.4. Gene ontology (GO) and Kyoto encyclopedia of genes and genomes (KEGG) enrichment analysis

Enrichment analysis allows to obtain important biological processes associated with DEGs. In this study, we conducted GO and KEGG functional enrichment analysis oncardiac aging-associated DEGs using the WebGestaltR (V0.4.4) package (18). The enriched GO terms and KEGG pathways were defined by *p* value < 0.05.

### 2.5. Weighted gene co-expression network analysis

To identify genes highly correlated with age, we used the WGCNA package (19) to identify gene modules in the GSE57338 cohort strongly correlated with age. The soft threshold *β* for module analysis was determined by analyzing the scale independence and average connectivity of the modules with different weighting factors. After we determined soft threshold, a scale-free topological distribution network was constructed, and the correlation matrix was converted into an adjacency matrix based on the Pearson correlation coefficient among genes and further into a topological overlap matrix (TOM). The similarity between genes (1-TOM) was calculated and genes with similar expression profiles were grouped into the same gene module using hierarchical clustering function and dynamic shear tree and a minimum size of 100. The gene modules most associated with age were determined using Pearson.

### 2.6. Protein-protein interaction networks (PPI) network analysis

The PPI network was constructed here through the STRING database (http://www.string-db.org/) (20) for co-expressed genes highly associated with age. The parameters were set as follows: low confidence: score <0.4; moderate: 0.4–0.7; height: >0.7, comprehensive score >0.4. Next, the PPI network was imported into Cytoscape software (http://cytoscape.org/, version 3.7.2) (21). The MCODE-based algorithm of metscape was used to find tightly connected proteomes in the target network and noting the biological function of each group. Finally, topological analysis was conducted on the PPI network, the degree of each gene was calculated, and genes with higher degree were selected as core genes.

### 2.7. Association between cardiac aging-associated hub genes and immunity

The intersection of MCODE and core genes was defined as the hub genes of cardiac aging. To clarify the immune status of each sample in the GSE35959, GSE141910, and GSE173608 cohorts, StromalScore, ImmuneScore, and ESTIMATEScore were calculated for all the samples using the ESTIMATE algorithm, and the abundance of 28 immune cells was determined by the ssGSEA method. We then calculated Pearson correlations between hub genes and each immune correlation score. Finally, the H.A. v7.4.symbols.gmt pathway of HALLMARK was obtained from GSEA website and its enrichment scores were calculated to evaluate the correlation between hub genes and pathway enrichment scores. The correlation heatmap was generated by the heatmap package (https://www.datanovia.com/en/lessons/heatmap-in-r-static-and-interactive-visualization/).

### 2.8. Molecular docking analysis

Sirolimus is an anti-aging drug (22), and we performed molecular docking simulations of Sirolimus with hub genes to select potential of hub genes for the treatment of cardiac aging. The molecular structures of hub genes proteins were download in the Protein Data Bank database (PDB, https://www1.rcsb.org/) and AlphaFold (https://alphafold.com/) database. Water molecules and proligands from target were removed by PyMOL 2.3.0. The Sirolimus molecular structures were obtained from the Pubchem database (https://pubchem.ncbi.nlm.nih.gov/). The conformation of Sirolimus was molecularly and mechanically optimized using Chem3D (version 2020, https://library.bath.ac.uk/chemistry-software/chem3d) software to obtain the optimal energy-minimized conformation of Sirolimus. The pretreated target protein molecules were hydrotreated using Auto Dock Tools1.5.6. The optimal conformation of Sirolimus was hydrogenated and the torsional bond was determined. POCASA protein active pocket online prediction tool was used to predict the protein active pocket, the docking range was set in the predicted active pocket and the docking range information was saved for formal docking. Auto Dock Vina v.1.2.0 was employed to conduct molecular simulation docking between target proteins and Sirolimus molecules using Lamarkian genetic algorithm and the semi-flexible. The exhaustiveness was set to 8, the maximum number of conformations output was set to 9. The binding free energy of Sirolimus to each hub gene protein was obtained.

### 2.9. Statistical analysis

All statistical analyses in this study were conducted in R software (version 4.1.1), PyMOL (version 2.3.0), and Chem3D (version 2020). And *p* < 0.05 was considered statistically significant. Sangerbox provided analytical assistance in this article (23).

## 3. Results

### 3.1. Correlation between age and immunity

**Figure 1:**
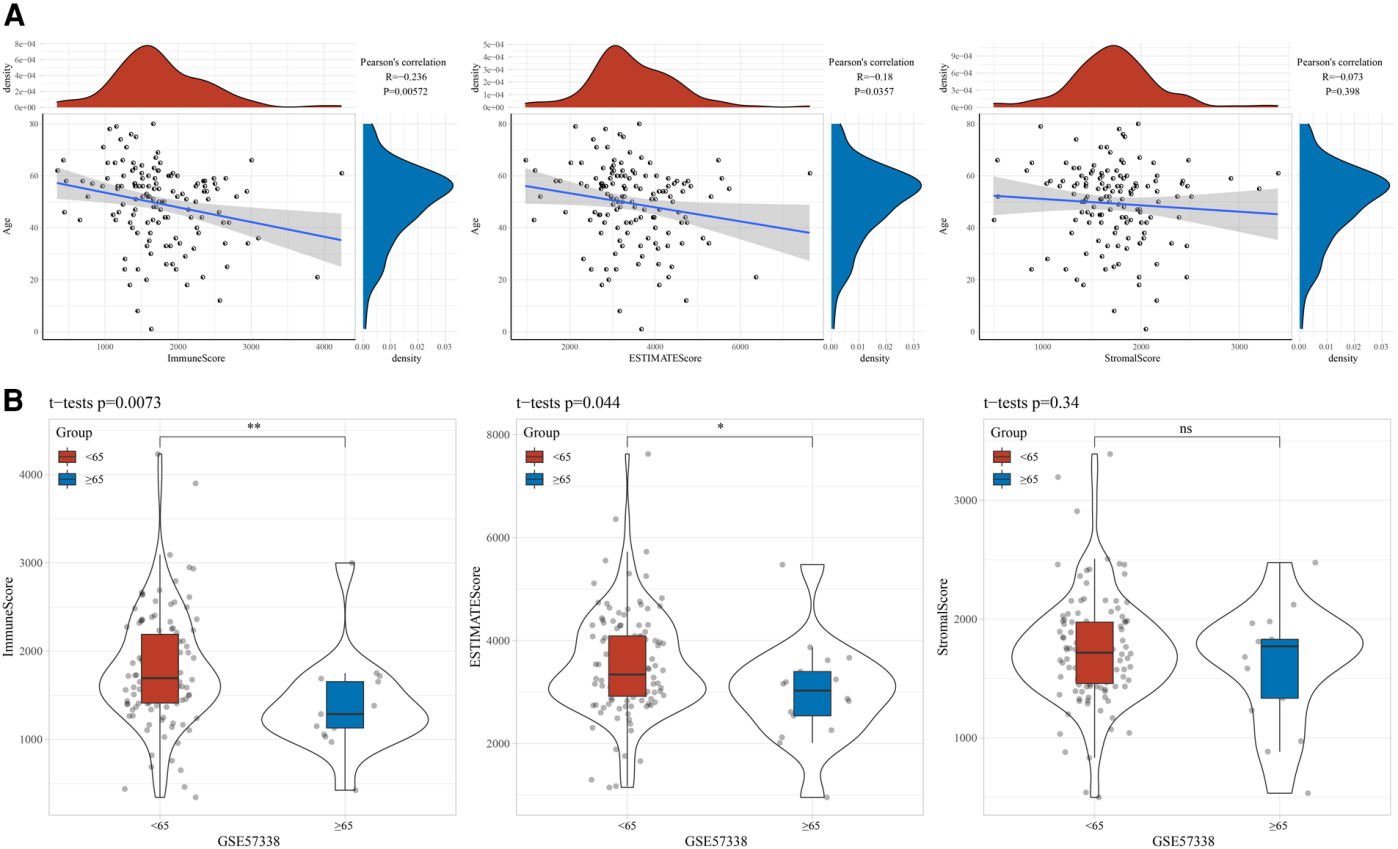
Correlation between age and immunity. (**A**) Correlation between age and stromalScore, immuneScore, ESTIMATEScore. (**B**) Differences in the distribution of stromalScore, immuneScore and ESTIMATEScore between age groups.

**Figure 2:**
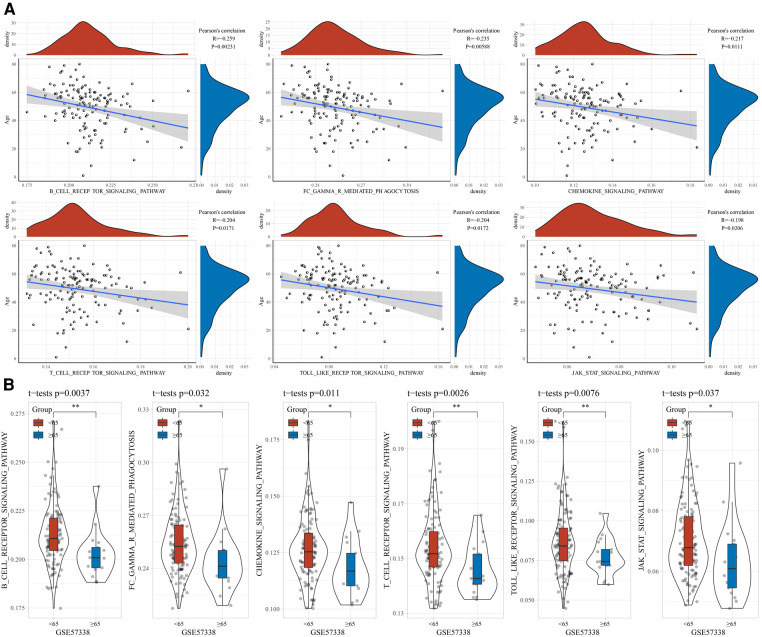
Correlation between age and immunity. (**A**) Association between age and immunity score. (**B**) Distribution of immunity scores between age groups.

To explore the association between age and immune status, we assessed the StromalScore, ImmuneScore, and ESTIMATEScore of the samples in the GSE57338 cohort using ESTAMATE software, and then calculated their Pearson correlation with age. It could be observed that age was negatively correlated with ImmuneScore and ESTIMATEScore, respectively (Figure 1A). The ImmuneScore and ESTIMATEScore of the young group were significantly higher than those of the elderly (Figure 1B). Naturally, the ssGSEA method was used to identify factors significantly associated with age. We found 73 pathways significantly associated with age, of which B_CELL_RECEPTOR_SIGNALING_PATHWAY (*R* = −0.259, *p* = 0.00231), FC_GAMMA_R_MEDIATED_PHAGOCYTOSIS (*R* = −0.235, *p* = 0.00588), CHEMOKINE_SIGNALING_PATHWAY (*R* = −0.217, *p* = 0.0111), T_CELL_ RECEPTOR_SIGNALING_PATHWAY (*R* = −0.204, *p* = 0.0171), TOLL_LIKE_RECEPTOR_SIGNALING_PATHWAY (*R* = −0.204, *p* = 0.0172), JAK_STAT_SIGNALING_PATHWAY (*R* = −0.198, *p* = 0.0206) were negatively correlated with age (Figure 2A). A violin plot showing the differences in these six pathway scores in the younger and older groups displayed that the six pathway scores were significantly higher in the younger group than in the older ones (Figure 2B).

### 3.2. Identification of cardiac aging-related DEGs

**Figure 3:**
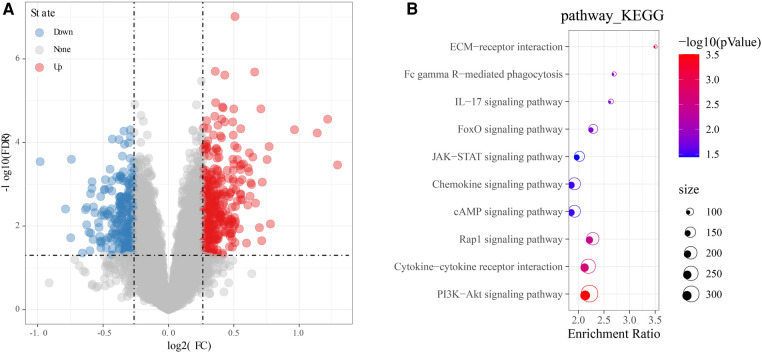
Identification and functional analysis of DEGs. (**A**) Volcano map of DEGs. (**B**) Bubble diagram of KEGG enrichment analysis of DEGs. Red means upregulated genes, and blue means downregulated genes, grey means there was no difference in gene expression.

In the GSE57338 cohort, a total of 606 DEGs, including 352 up-regulated genes and 254 down-regulated genes, were identified by differential analysis on the older and younger groups under the screening threshold of *p* < 0.05 and |log2FC| > 1.2 (Figure 3A). GO and KEGG enrichment analysis revealed that these 606 DEGs were mainly enriched in Fc gamma R-mediated phagocytosis, IL-17 signaling pathway, Chemokine signaling pathway, Cytokine-cytokine receptor interaction, ECM-receptor interaction, FoxO signaling pathway, JAK-STAT signaling pathway, cAMP signaling pathway, PI3K-Akt signaling pathway (Figure 3B). Most of these pathways can be found to be associated with chemokine signaling in immune response.

### 3.3. WGCNA analysis

**Figure 4:**
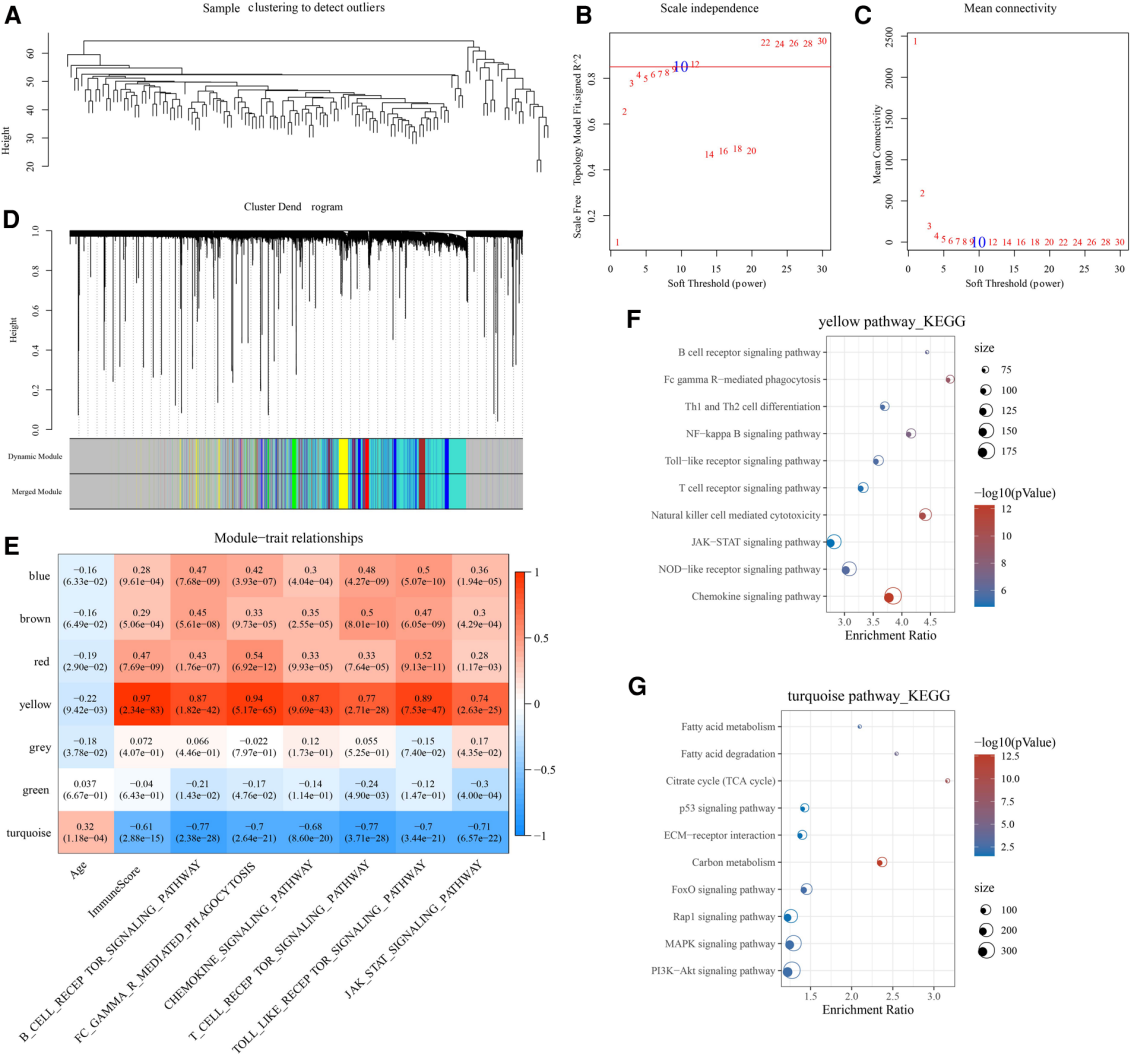
WGCNA construction. (**A**) Hierarchical clustering tree for each sample in the GSE57338 cohort. (**B**) The scale-free fit index for various soft-thresholding powers. (**C**) The mean connectivity for various soft-thresholding powers. (**D**) Genes are divided into different modules by hierarchical clustering, and different colors represent different modules. (**E**) Correlation of module eigenvectors of modules with age and immunity. (**F**) Functional annotation of yellow module genes. (**G**) Functional annotation of turquoise module genes.

A co-expression network was constructed in the GSE57338 cohort to identify age-associated gene modules. Specifically, the samples were first clustered and outlier samples were excluded, and the sample clustering map is shown in Figure 4A. In this study, we found that the co-expression network at this time was consistent with the scale-free network when the power *β* = 10 (Figures 4B,C). Similar gene modules are merged by dynamic shear tree algorithm (Figure 4D). Furthermore, we analyzed the Pearson correlation of each module with immune-related scores, and found that the yellow module was significantly negatively correlated with age (*R* = −0.22, *p *< 0.05), and positively correlated with ImmuneScore (*R* = 0.97, *p *< 0.05), B_CELL_RECEPTOR_SIGNALING_PATHWAY (*R* = 0.87, *p *< 0.05), FC_GAMMA_R_MEDIATED_PHAGOCYTOSIS (*R* = 0.94, *p *< 0.05), CHEMOKINE_SIGNALING_PATHWAY (*R* = 0.87, *p *< 0.05), T_CELL_RECEPTOR_SIGNALING_PATHWAY (*R* = 0.77, *p *< 0.05), TOLL_LIKE_RECEPTOR_SIGNALING_PATHWAY (*R* = 0.89, *p *< 0.05), JAK_STAT_SIGNALING_PATHWAY (*R* = 0.74, *p *< 0.05). The turquoise module was significantly positively correlated with age (*R* = 0.32, *p *< 0.05) and significantly negatively correlated with ImmuneScore (*R* = −0.61, *p *< 0.05), B_CELL_RECEPTOR_SIGNALING_PATHWAY (*R* = −0.77, *p *< 0.05), FC_GAMMA_R_MEDIATED_PHAGOCYTOSIS (*R* = −0.7, *p *< 0.05), CHEMOKINE_SIGNALING_PATHWAY (*R* = −0.68, *p *< 0.05), T_CELL_RECEPTOR_SIGNALING_PATHWAY (*R* = −0.77, *p *< 0.05), TOLL_LIKE_RECEPTOR_SIGNALING_PATHWAY (*R* = −0.7, *p *< 0.05), JAK_STAT_SIGNALING_PATHWAY (*R* = −0.71, *p *< 0.05) (Figure 4E). Finally, we analyzed the biological functions of genes in the yellow and turquoise modules. It was observed that the yellow module genes were mainly enriched in immune-related pathways such as T cell receptor signaling pathway, B cell receptor signaling pathway, and Natural killer cell mediated cytotoxicity (Figure 4F). The turquoise module genes were mainly enriched in PI3K-Akt signaling pathway, p53 signaling pathway, ECM-receptor interaction and other related pathways (Figure 4G). Therefore, the yellow and turquoise modules were chosen for subsequent analysis.

### 3.4. PPI network

**Figure 5:**
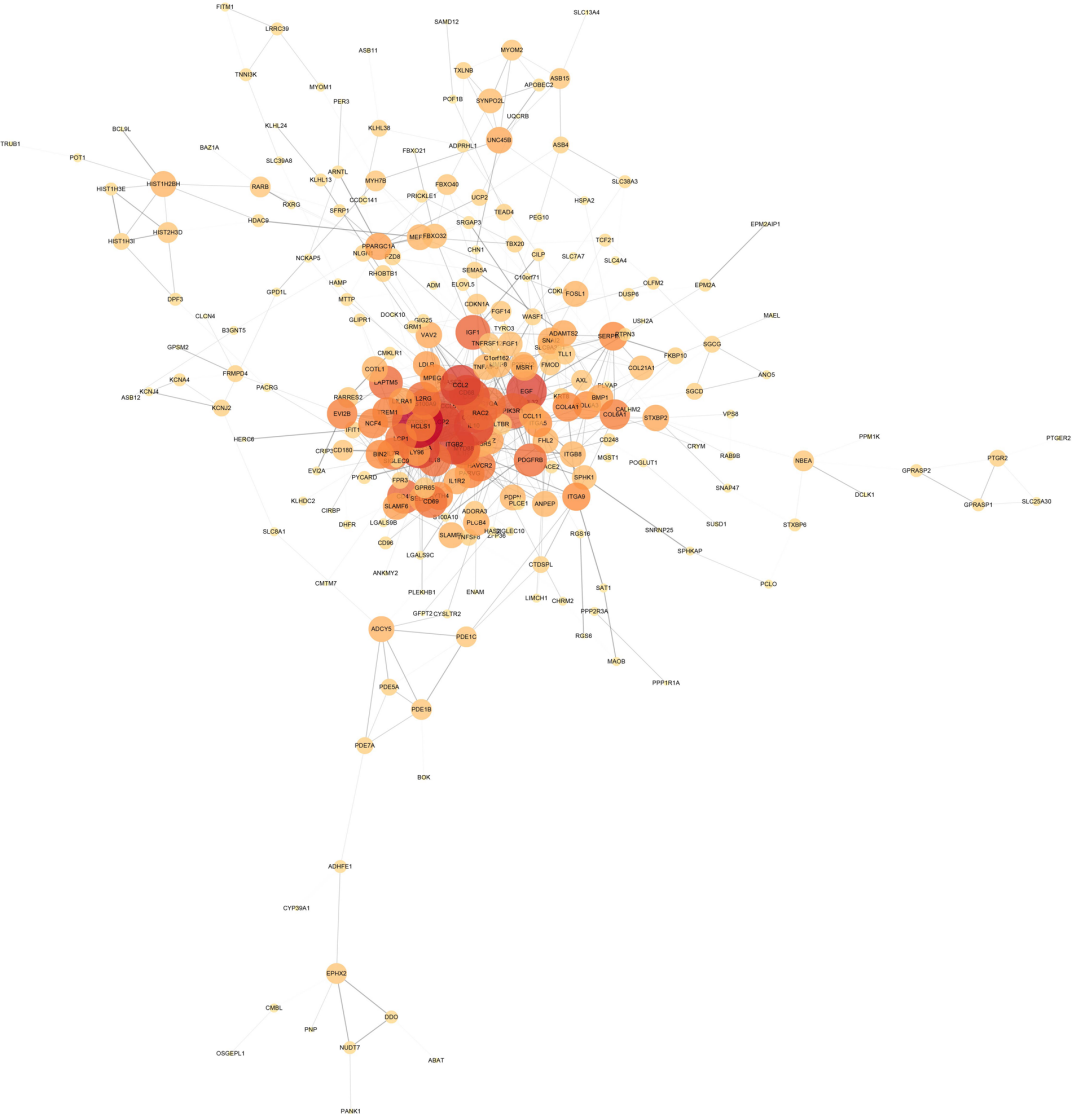
PPI network diagram, the darker the color and the larger the circle, the more important the gene; the thickness of the line represents the strength of the binding between genes.

**Figure 6:**
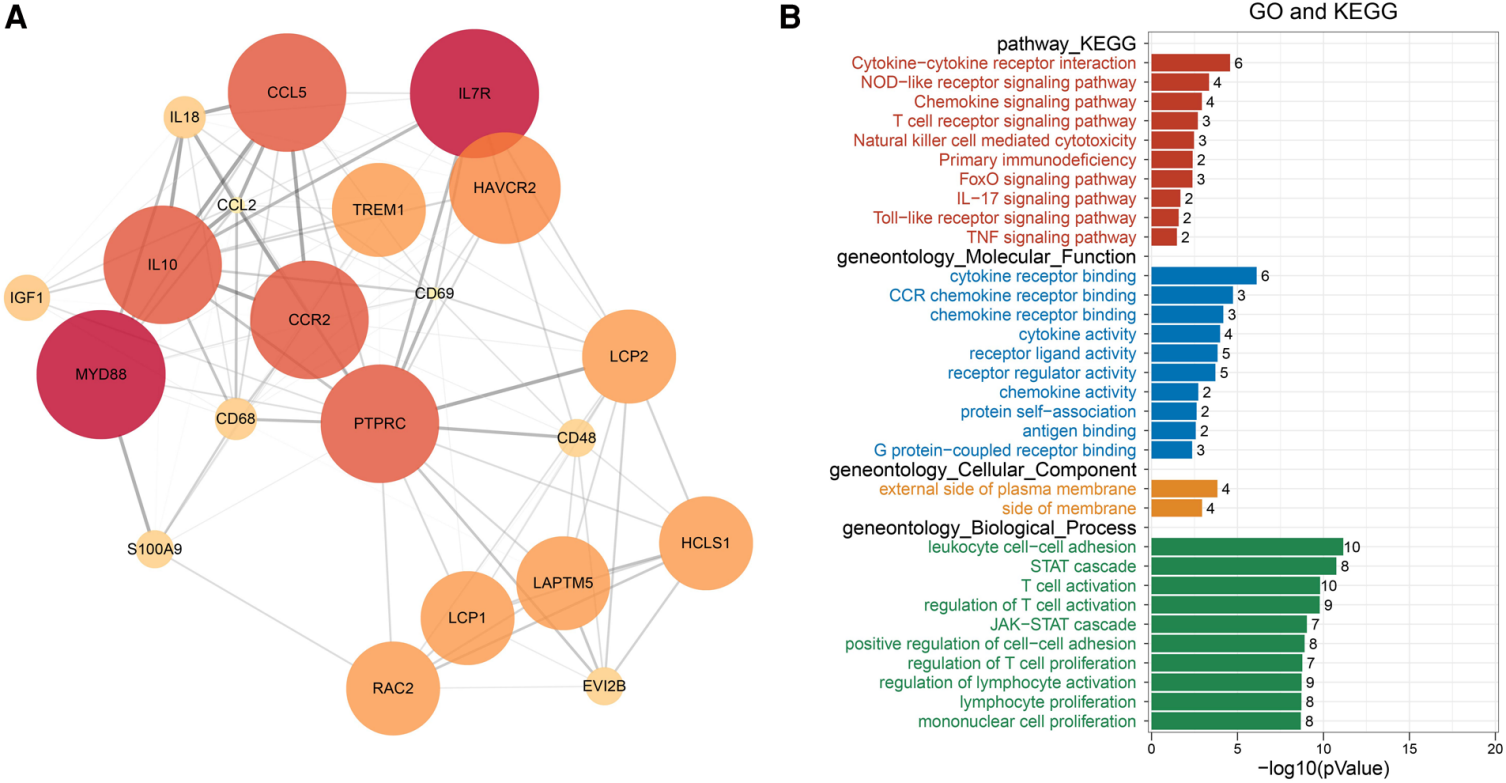
PPI network module analysis by MCODE. (**A**) Module-based network analysis of potential genes. (**B**) Functional annotation of MCODE module 1 gene.

The intersection of the yellow module, the turquoise module and 606 DEGs had a total of 380 genes, which were mapped in the String database to construct a PPI network. The nodes with fewer edges in the PPI network were eliminated, and 255 nodes were finally retained for subsequent analysis (Figure 5). Nodes in PPI were analyzed by metscape. The MCODE plugin in cytoscape was used to find tightly linked proteomes in the network (Figure 6A). Four functional modules were extracted from 255 nodes, and we performed functional enrichment analysis on the genes in MCODE module 1. The results showed that the genes in MCODE module 1 were mainly involved in Cytokine-cytokine receptor interaction, Chemokine signaling pathway, T cell receptor signaling pathway, Natural killer cell mediated cytotoxicity, IL-17 signaling pathway, Toll-like receptor signaling pathway and other biological writing functions (Figure 6B). These results indicated that genes highly associated with these age-related factors were also closely related to immune function. Finally, we calculated the degree of 255 nodes in the PPI network, from which we selected the top 15 genes as the potential core genes (Table 1). These genes may be hub genes in the aging process of the cardiac aging.

**Table 1:** Topological parameters of core genes in the PPI network.

Numbers	Gene	Degree	Betweenness centrality	Closeness centrality
1	PTPRC	52	0.12872569	0.36705202
2	EGF	33	0.11947336	0.35425384
3	CCL2	33	0.07109627	0.34370771
4	ITGB2	33	0.06433343	0.35034483
5	FCGR3A	33	0.03356093	0.32987013
6	IL10	32	0.0765978	0.34324324
7	LCP2	31	0.03237325	0.32439336
8	CCL5	27	0.0224443	0.32315522
9	CCR2	27	0.01555626	0.32439336
10	PIK3R1	24	0.14734879	0.35825106
11	CD68	24	0.01401788	0.32439336
12	IGF1	23	0.14235186	0.35674157
13	CD48	22	0.01491111	0.3020214
14	RAC2	21	0.05313282	0.32480818
15	IL2RG	21	0.03304713	0.32816537

### 3.5. Correlation between hub genes and immunity

**Figure 7:**
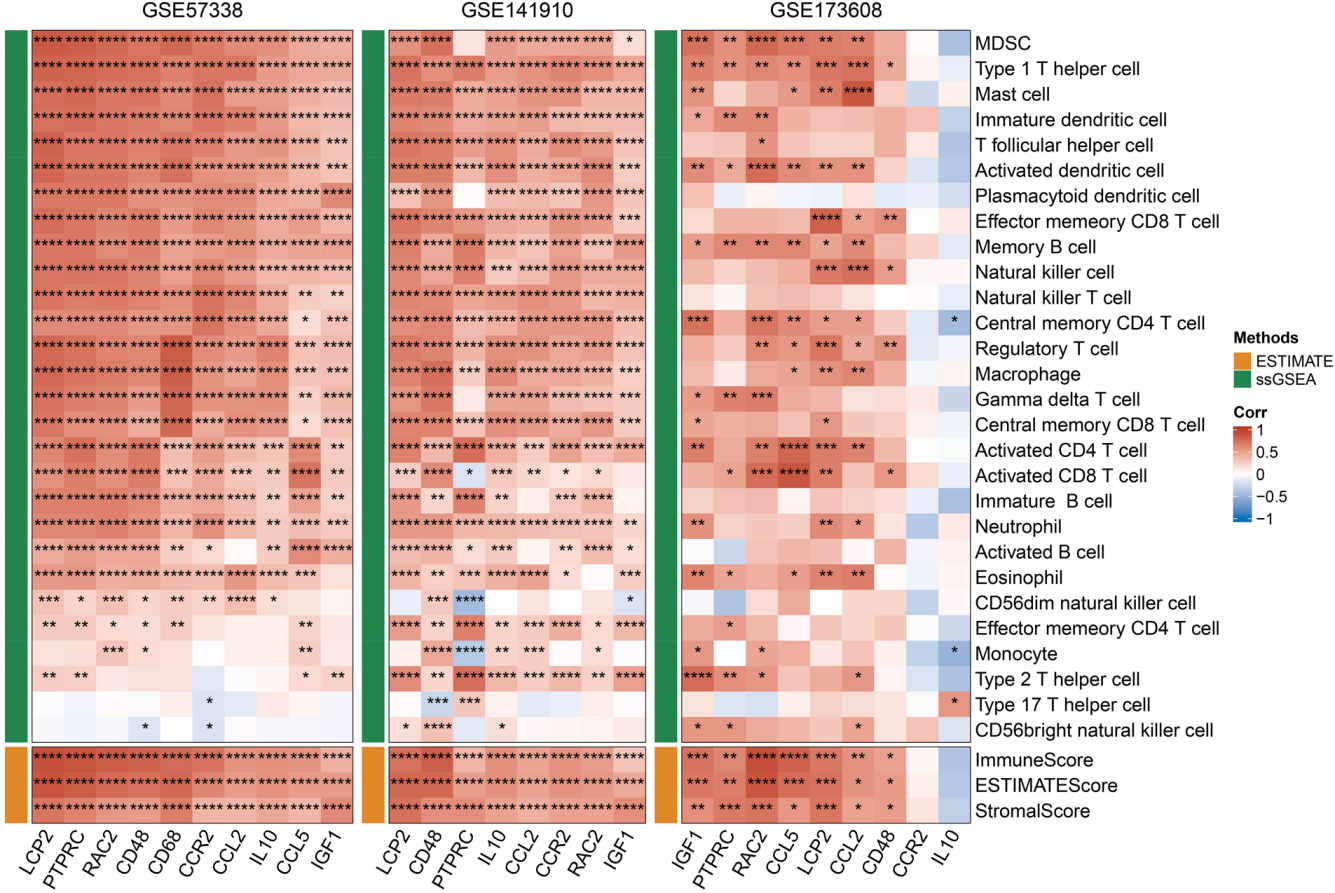
Correlation between hub genes and immunity, red represents positive correlation, blue represents negative correlation, *p < 0.05, **p < 0.01, ***p < 0.001, ****p < 0.0001.

After the intersection of MCODE module gene and 15 potential core genes, a total of 10 genes including LCP2, PTPRC, RAC2, CD48, CD68, CCR2, CCL2, IL10, CCL5 and IGF1 were obtained. To assess the association between the 10 hub genes and immunity, we first calculated the StromalScore, ImmuneScore, ESTIMATEScore and ssGSEA scores of 28 kinds of immune cells in the GSE35959, GSE141910 and GSE173608 cohorts. Through Pearson correlation analysis, we found that the 10 hub genes showed a positive correlation with most immune scores, and that they were significantly positively correlated with Type 1 T helper cell Immature dendritic cell and immature dendritic cell (Figure 7). Considering telomere shortening is an important feature of aging, the association analysis of 10 hub genes and telomerase genes in 3 datasets indicated that 10 hub genes were associated to telomerase genes varying degrees (Supplementary Figure S1).

### 3.6. Correlation between hub genes and pathways

**Figure 8:**
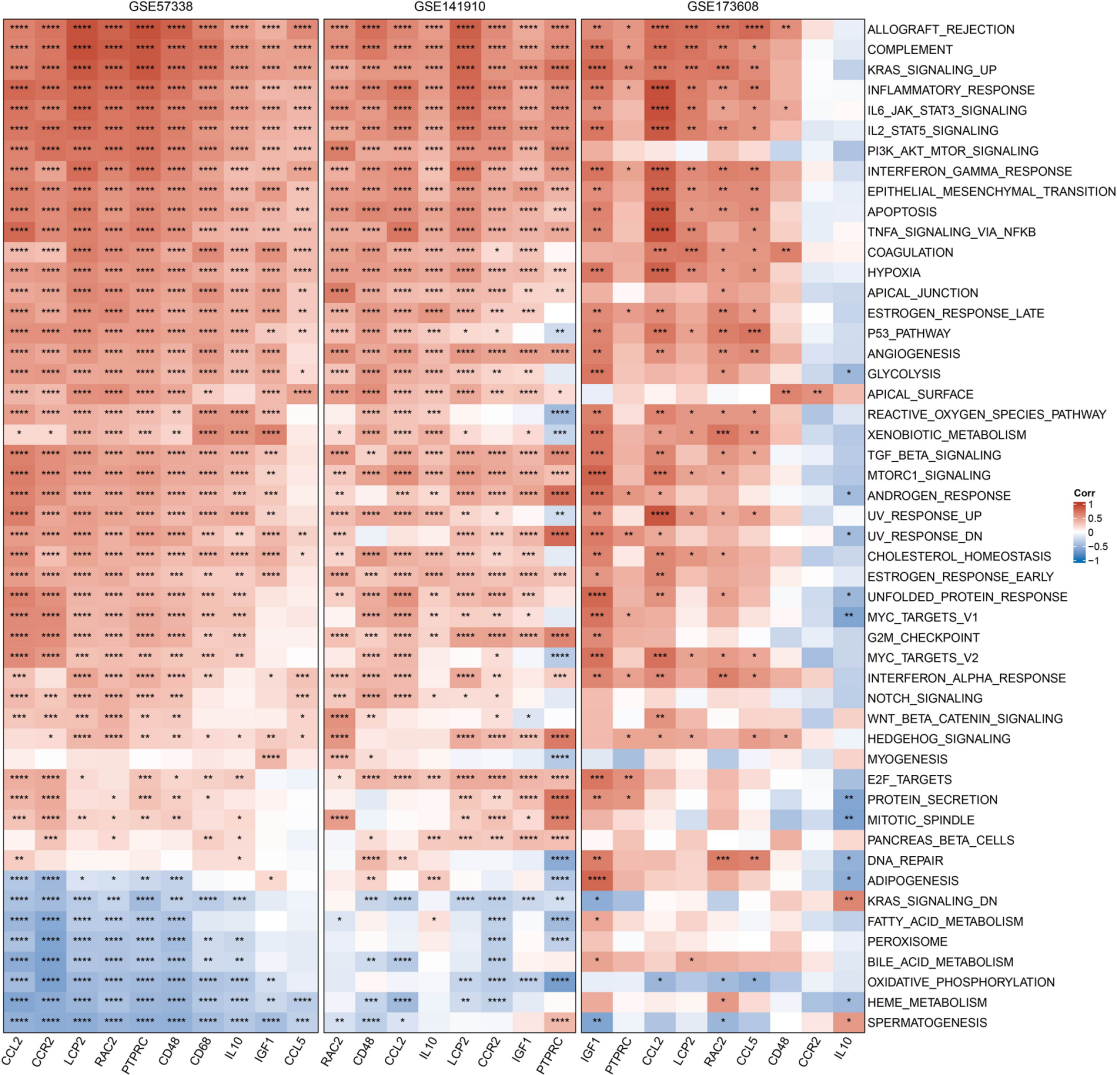
Correlation between hub genes and pathways, red represents positive correlation, blue represents negative correlation, *p < 0.05, **p < 0.01, ***p < 0.001, ****p < 0.0001.

We obtained the h.all.v7.4.symbols.gmt pathway from HALLMARK in the GSEA website and used the ssGSEA method to calculate these pathway scores in the GSE35959, GSE141910, and GSE173608 cohorts. We then calculated Pearson correlation coefficients between the 10 hub genes and pathway scores. The heat map showed that the trend of the 10 hub genes in the GSE35959, GSE141910, and GSE173608 cohorts was generally consistent, and that they were mainly positively correlated with IL6_JAK_STAT3_SIGNALING, P53_PATHWAY, EPITHELIAL_MESENCHYMAL_TRANSITION, ALLOGRAFT_REJECTION but negatively correlated with OXIDATIVE_PHOSPHORYLATION, FATTY_ACID_METABOLISM (Figure 8).

### 3.7. Molecular docking simulation

**Figure 9:**
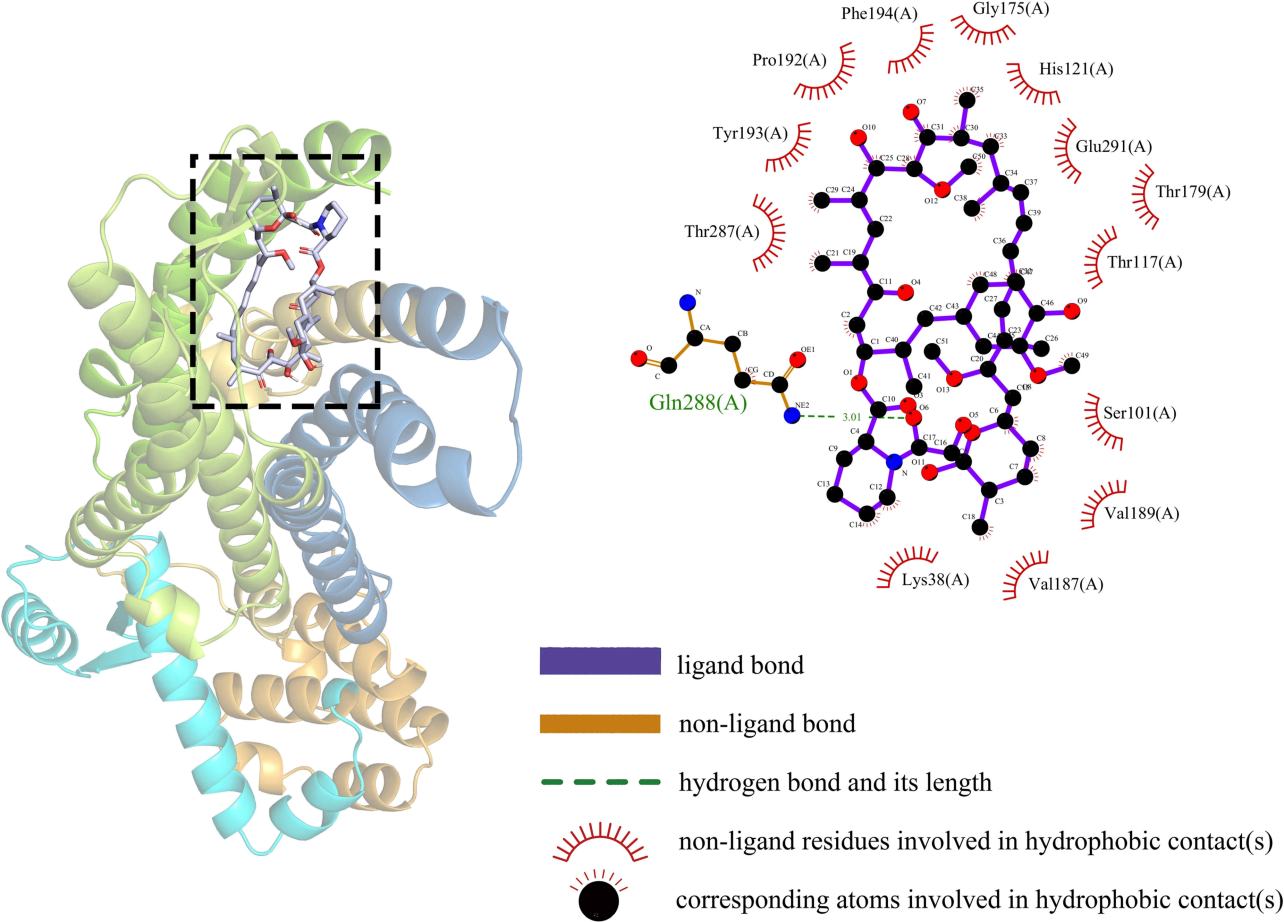
Schematic diagram of sirolimus-CCR2 molecular docking.

In this study, the binding stability of Sirolimus to 10-hub genes was assessed using molecular docking techniques to identify the optimal cardiac aging genes. Generally speaking, a binding energy less than −5 kcal/mol indicates an excellent binding and less than −7 kcal/mol indicates a strong binding. The molecular docking results were shown in Table 2, from which it could be observed that there were strong binding interactions between Sirolimus and all 10-hub genes, with the strongest direct in Sirolimus-CCR2 binding. The 3D schematic of Sirolimus-CCR2 binding is displayed in Figure 9. The 2D schematic diagram showed that Sirolimus formed a hydrogen bond with the amino acid residue Gln-288 of the A chain of the CCR2 protein and a hydrophobic interaction with 13 amino acid residues such as THR-287, TYR-19 and PRO-192 (Figure 9).

**Table 2:** Molecular docking results of sirolimus and 10-hub genes.

Name	Center (x, y, z)	Size (x × y × z)	Energy (kcal/mol)	RMSD
CCL2	12, −29, −20	27 × 27 × 27	−7.2	7.226
CCL5	−6, 15, −27	27 × 27 × 27	−7.2	7.037
CCR2	6, 21, 155	27 × 27 × 27	−10.5	6.879
CD48	25, 1, −24	27 × 27 × 27	−8.3	7.238
CD68	7, −9, −1	27 × 27 × 27	−8.6	7.407
IGF1	3, −2, −28	27 × 27 × 27	−8.4	6.669
IL10	8, 48, 37	27 × 27 × 27	−9.8	6.363
LCP2	−16, −5, −3	27 × 27 × 27	−8.9	6.426
PTPRC	6, −4, 33	27 × 27 × 27	−8.0	6.817
RAC2	−9, −30, 22	27 × 27 × 27	−9.8	6.948

### 3.8. Construction of the diagnosis model using 10 hub genes

**Figure 10:**
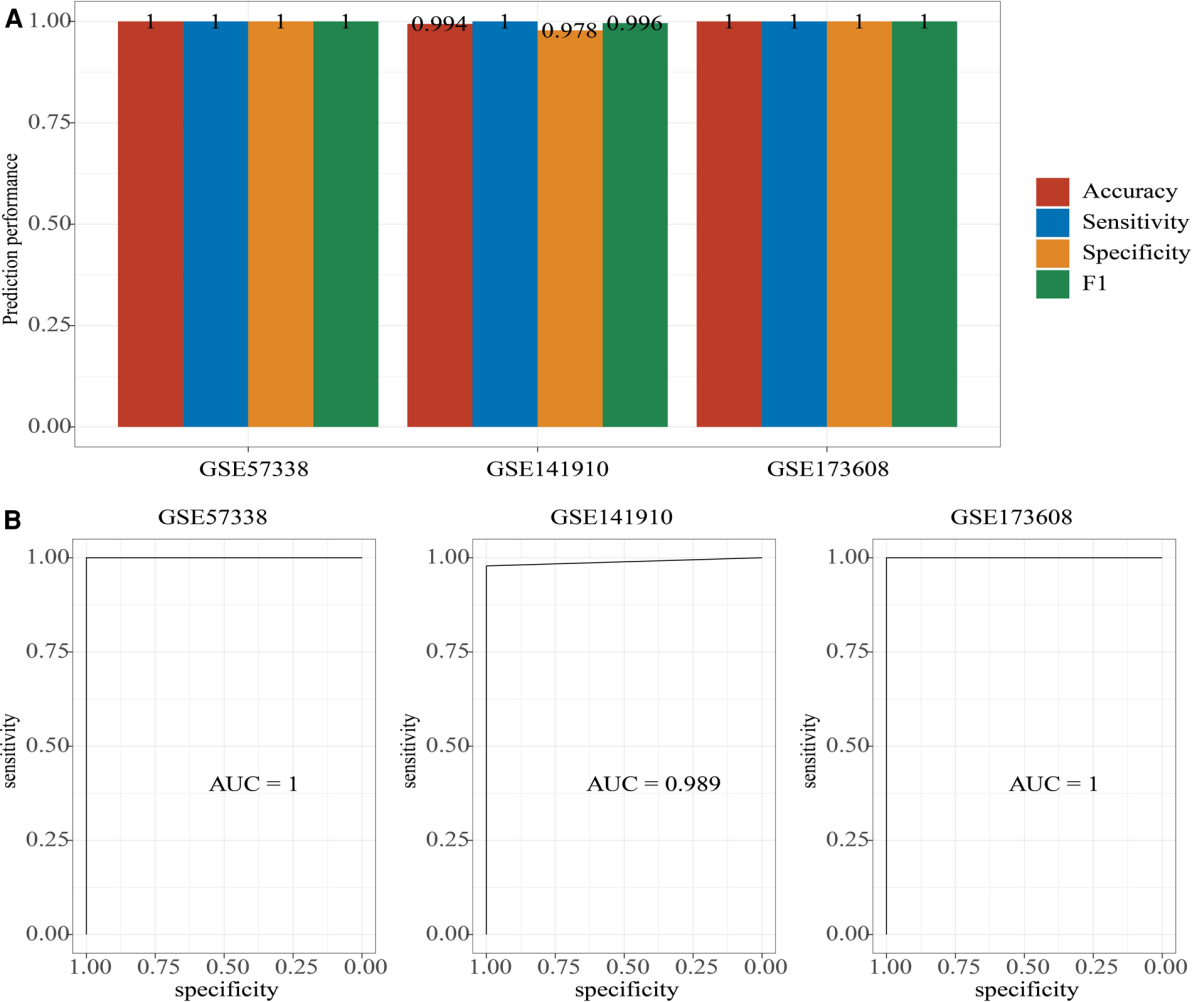
Construction of a diagnosis model. (**A**) The accuracy, sensitivity, specificity, and F1 of diagnosis model in GSE57338 dataset, GSE141910 dataset and GSE173608 dataset. (**B**) The AUC of diagnosis model in GSE57338 dataset, GSE141910 dataset and GSE173608 dataset.

The 10 hub genes were used to construct diagnosis model in GSE57338 dataset using Xgboost package (eta = 0.3, gama = 0.001, max_depth = 3, subsample = 0.7, colsample_bytree = 0.4, num_class = 2, objective = “multi:softprob”, nrounds = 1,000), and validated in GSE141910 dataset and GSE173608 dataset. The Accuracy, sensitivity, specificity and F1 of the diagnosis model in GSE57338 dataset and GSE173608 dataset were both 1, and in GSE141910 dataset were respectively 0.994, 1, 0.978 and 0.996 (Figure 10A). In addition, the AUC were 1, 0.989, and 1 respectively in GSE57338 dataset, GSE141910 dataset and GSE173608 dataset (Figure 10B). The analysis data showed that the diagnosis model could identified Cardiac aging from samples.

## 4. Discussion

Given the lack of age-related biomarkers of cardiac aging, it is necessary to identify potential molecular mechanisms and hub genes in cardiac aging through emerging technologies. Here, we identified age-associated hub genes of cardiac aging by analyzing transcriptome sequencing data from different age-stratified populations, and primarily explored the potential mechanisms associated with cardiac aging.

The 10 identified genes included LCP2, PTPRC, RAC2, CD48, CD68, CCR2, CCL2, IL10, CCL5 and IGF1. We also found that most of the 10-hub genes were positively correlated with immune scores and immune-related pathways. Previous studies have confirmed a close relationship between these genes and immunity. Lymphocyte Cytosolic Protein 2 (LCP2) encodes the bridging protein SLP76, and Siggs et al. (24) showed that a splice variant in LCP2 decreases SLP76 levels, triggering immune dysregulation and ultimately excessive production of pro-inflammatory cytokines and autoantibodies. Protein Tyrosine Phosphatase Receptor Type C (PTPRC), also known as CD45, was found in one study to maintain its expression in CD45-derived cells, and the percentage of CD45-positive extracardiac cells located within endothelial cells as well as in the interstitial region of heart valve structures increases with age (25). Ning et al. (26) showed that suppression of RAC2 expression reduced isoproterenol-induced cardiac injury and fibrosis. cd48 promoted interactions between activated lymphocytes and was involved in regulatory T cell activation (27). Zawia et al. (28) reported a decrease in CD68^+^ macrophages in mice correlation with the development of PAH.

Jung et al. (29) pointed out that IL10 plays an essential role in the cardiac remodeling process after myocardial infarction. When IL-10 is injected into mice with myocardial infarction, fibroblast activation (proliferation, migration and collagen production) could be significantly observed under the influence of macrophage M2. Young et al. (30) demonstrated that the decreased expression level of IGF1 in the bone marrow microenvironment would stimulate the aging of hematopoietic stem cells, and that the aging trend of patients could be improved by stimulating the recovery of IGF1 expression of hematopoietic stem cells. In this study, we found that these genes were highly connected in PPI network and WGCNA module, suggesting that they might play an essential role in cardiac aging. Evidence from previous studies has confirmed a close relationship between these genes and immune or cellular senescence. Therefore, we reasonably speculated that they may be major evidence to explain the mechanisms of cardiac aging.

Robbie et al. (31) noted that aging is associated with upregulation of proinflammatory-related signaling pathways caused by the CCL2-CCR2 axis during retinal aging, and that CCL2-driven myeloid cell recruitment and CNV attenuation severity increases with age. Moreover, Robbie et al. also noted that similar molecular mechanisms may be associated with other age-related inflammatory diseases. In this study, CCL2 and CCR2 were the hub genes associated with cardiac aging, but the connection between the CCL2-CCR2 axis and cardiac aging has not yet been elucidated, which could be a breakthrough for future research. Interestingly, molecular docking analysis showed that the anti-aging drug Sirolimus had excellent and stable binding to CCR2, the binding of Sirolimus to CCR2 target proteins was likely to exert corresponding pharmacological effects. Sirolimus was found to extend the life span of mice in a study by Harrison et al. (32). Studies have shown that Sirolimus can reduce mitochondrial reactive oxygen species and inhibit cardiac hypertrophy and cardiac ageing through the inhibition of mTORC1 (14, 33). Das et al. have shown that Sirolimus can reverse the metabolic changes associated with ageing and thereby exert a cardioprotective effect in experimental models of cardiac ageing (34). It was experimentally confirmed that Sirolimus extended the life span of male and female mice by 9% and 14%, respectively. However, published papers indicated that Sirolimus is an mTOR inhibitor that reduces the rate of cellular senescence through inhibiting the mTOR pathway, which in turn ameliorates aging-related diseases (35). There were no reported studies on Sirolimus action on CCR2 to inhibit senescence, which may be a new potential molecular mechanism of effect.

In summary, this report applied bioinformatics such as WGCNA to mine 10 cardiac aging-related hub genes, which may provide new insights for elucidating the risk of cardiac aging. Apart from our systematic bioinformatics analyses, the present study also has limitations. Firstly, this study was based on bioinformatics approach using a calculator and other related devices as a preliminary data analysis, and the specific biological functions of the 10-hub genes will have to be explored at the cellular, molecular, and animal levels, as well as clinical shape. Secondly, Sirolimus is an anti-aging drug but is not currently used in treating cardiac aging-related diseases, and its potential relationship with CCR2 should be further explored. Therefore, conducting comprehensive and systematic *in vivo* and *in vitro* assays to explore more in-depth molecular mechanisms is our subsequent key research targets.

## 5. Conclusion

In the present report, we identified 10 hub genes associated with cardiac aging and systematically elucidated the correlation between these genes and immunity. Our study revealed that cardiac aging was correlated with immune system activity, and that CCR2 may be a potential core target in cardiac aging. The Sirolimus-CCR2 interaction relationship provided an important scientific basis for elucidating cardiac aging-related gene functions and may help to elucidate aging-related mechanisms in human life.

## Data Availability

The original contributions presented in the study are included in the article/**Supplementary Material**, further inquiries can be directed to the corresponding authors.

## References

[1] Magenta A Lorde R Syed SB Capogrossi MC Puca A Madeddu P. Molecular therapies delaying cardiovascular aging: disease- or health-oriented approaches. Vasc Biol. (2020) 2(1):R45–R58. 10.1530/VB-19-0029 32923974 PMC7439942

[2] Zhang Q Wang L Wang S Cheng H Xu L Pei G Signaling pathways and targeted therapy for myocardial infarction. Signal Transduct Target Ther. (2022) 7(1):78. 10.1038/s41392-022-00925-z 35273164 PMC8913803

[3] Roth GA Mensah GA Fuster V. The global burden of cardiovascular diseases and risks: a compass for global action. J Am Coll Cardiol. (2020) 76(25):2980–1. 10.1016/j.jacc.2020.11.021 33309174

[4] Roth GA Mensah GA Johnson CO Addolorato G Ammirati E Baddour LM Global burden of cardiovascular diseases and risk factors, 1990-2019: update from the GBD 2019 study. J Am Coll Cardiol. (2020) 76(25):2982–3021. 10.1016/j.jacc.2020.11.010 33309175 PMC7755038

[5] Chen MS Lee RT Garbern JC. Senescence mechanisms and targets in the heart. Cardiovasc Res. (2022) 118(5):1173–87. 10.1093/cvr/cvab161 33963378 PMC8953446

[6] Deng M Yin Y Zhang Q Zhou X Hou G. Identification of inflammation-related biomarker lp-PLA2 for patients with COPD by comprehensive analysis. Front Immunol. (2021) 12:670971. 10.3389/fimmu.2021.670971 34093570 PMC8176901

[7] Chen S Yang D Lei C Li Y Sun X Chen M Identification of crucial genes in abdominal aortic aneurysm by WGCNA. PeerJ. (2019) 7:e7873. 10.7717/peerj.7873 31608184 PMC6788446

[8] Zhao X Sui Y Ruan X Wang X He K Dong W A deep learning model for early risk prediction of heart failure with preserved ejection fraction by DNA methylation profiles combined with clinical features. Clin Epigenetics. (2022) 14(1):11. 10.1186/s13148-022-01232-8 35045866 PMC8772140

[9] Qu LH Luo WJ Yan ZG Liu WP. FAM171B as a novel biomarker mediates tissue immune microenvironment in pulmonary arterial hypertension. Mediators Inflamm. (2022) 2022:1878766. 10.1155/2022/1878766 36248192 PMC9553458

[10] Liu X Xu S Li Y Chen Q Zhang Y Peng L. Identification of CALU and PALLD as potential biomarkers associated with immune infiltration in heart failure. Front Cardiovasc Med. (2021) 8:774755. 10.3389/fcvm.2021.774755 34926621 PMC8671636

[11] Nakagawa H Fujita M. Whole genome sequencing analysis for cancer genomics and precision medicine. Cancer Sci. (2018) 109(3):513–22. 10.1111/cas.13505 29345757 PMC5834793

[12] Yan M Sun S Xu K Huang X Dou L Pang J Cardiac aging: from basic research to therapeutics. Oxid Med Cell Longev. (2021) 2021:9570325. 10.1155/2021/9570325 33777324 PMC7969106

[13] Martínez-Cisuelo V Gómez J García-Junceda I Naudí A Cabré R Mota-Martorell N Rapamycin reverses age-related increases in mitochondrial ROS production at complex I, oxidative stress, accumulation of mtDNA fragments inside nuclear DNA, and lipofuscin level, and increases autophagy, in the liver of middle-aged mice. Exp Gerontol. (2016) 83:130–8. 10.1016/j.exger.2016.08.002 27498120

[14] Xia Y Sun M Xie Y Shu R. mTOR inhibition rejuvenates the aging gingival fibroblasts through alleviating oxidative stress. Oxid Med Cell Longev. (2017) 2017:6292630. 10.1155/2017/6292630 28804534 PMC5540269

[15] Yoshihara K Shahmoradgoli M Martinez E Vegesna R Kim H Torres-Garcia W Inferring tumour purity and stromal and immune cell admixture from expression data. Nat Commun. (2013) 4:2612. 10.1038/ncomms3612 24113773 PMC3826632

[16] Subramanian A Tamayo P Mootha VK Mukherjee S Ebert BL Gillette MA Gene set enrichment analysis: a knowledge-based approach for interpreting genome-wide expression profiles. Proc Natl Acad Sci U S A. (2005) 102(43):15545–50. 10.1073/pnas.0506580102 16199517 PMC1239896

[17] Ritchie ME Phipson B Wu D Hu Y Law CW Shi W Limma powers differential expression analyses for RNA-sequencing and microarray studies. Nucleic Acids Res. (2015) 43(7):e47. 10.1093/nar/gkv007 25605792 PMC4402510

[18] Liao Y Wang J Jaehnig EJ Shi Z Zhang B. Webgestalt 2019: gene set analysis toolkit with revamped UIs and APIs. Nucleic Acids Res. (2019) 47(W1):W199–205. 10.1093/nar/gkz401 31114916 PMC6602449

[19] Langfelder P Horvath S. WGCNA: an R package for weighted correlation network analysis. BMC Bioinform. (2008) 9:559. 10.1186/1471-2105-9-559 PMC2631488 19114008

[20] Szklarczyk D Gable AL Nastou KC Lyon D Kirsch R Pyysalo S The STRING database in 2021: customizable protein-protein networks, and functional characterization of user-uploaded gene/measurement sets. Nucleic Acids Res. (2021) 49(D1):D605–D12. 10.1093/nar/gkaa1074 33237311 PMC7779004

[21] Shannon P Markiel A Ozier O Baliga NS Wang JT Ramage D Cytoscape: a software environment for integrated models of biomolecular interaction networks. Genome Res. (2003) 13(11):2498–504. 10.1101/gr.1239303 14597658 PMC403769

[22] Partridge L Fuentealba M Kennedy BK. The quest to slow ageing through drug discovery. Nat Rev Drug Discov. (2020) 19(8):513–32. 10.1038/s41573-020-0067-7 32467649

[23] Shen W Song Z Xiao Z Huang M Shen D Gao P Sangerbox: a comprehensive, interaction-friendly clinical bioinformatics analysis platform. iMeta. (2022) 1(3):e36. 10.1002/imt2.36 PMC10989974 38868713

[24] Siggs OM Miosge LA Daley SR Asquith K Foster PS Liston A Quantitative reduction of the TCR adapter protein SLP-76 unbalances immunity and immune regulation. J Immunol. (2015) 194(6):2587–95. 10.4049/jimmunol.1400326 25662996 PMC4355390

[25] Anstine LJ Horne TE Horwitz EM Lincoln J. Contribution of extra-cardiac cells in murine heart valves is age-dependent. J Am Heart Assoc. (2017) 6(10):e007097. 10.1161/JAHA.117.007097 29054843 PMC5721893

[26] Ning BB Zhang Y Wu DD Cui JG Liu L Wang PW Luteolin-7-diglucuronide attenuates isoproterenol-induced myocardial injury and fibrosis in mice. Acta Pharmacol Sin. (2017) 38(3):331–41. 10.1038/aps.2016.142 28112175 PMC5342667

[27] Lissina A Ambrozak DR Boswell KL Yang W Boritz E Wakabayashi Y Fine-tuning of CD8(+) T-cell effector functions by targeting the 2B4-CD48 interaction. Immunol Cell Biol. (2016) 94(6):583–92. 10.1038/icb.2016.17 26860368

[28] Zawia A Arnold ND West L Pickworth JA Turton H Iremonger J Altered macrophage polarization induces experimental pulmonary hypertension and is observed in patients with pulmonary arterial hypertension. Arterioscler Thromb Vasc Biol. (2021) 41(1):430–45. 10.1161/ATVBAHA.120.314639 33147993 PMC7752239

[29] Jung M Ma Y Iyer RP DeLeon-Pennell KY Yabluchanskiy A Garrett MR IL-10 improves cardiac remodeling after myocardial infarction by stimulating M2 macrophage polarization and fibroblast activation. Basic Res Cardiol. (2017) 112(3):33. 10.1007/s00395-017-0622-5 28439731 PMC5575998

[30] Young K Eudy E Bell R Loberg MA Stearns T Sharma D Decline in IGF1 in the bone marrow microenvironment initiates hematopoietic stem cell aging. Cell Stem Cell. (2021) 28(8):1473–82 e7. 10.1016/j.stem.2021.03.017 33848471 PMC8349778

[31] Robbie SJ Georgiadis A Barker SE Duran Y Smith AJ Ali RR Enhanced Ccl2-Ccr2 signaling drives more severe choroidal neovascularization with aging. Neurobiol Aging. (2016) 40:110–9. 10.1016/j.neurobiolaging.2015.12.019 26973110

[32] Harrison DE Strong R Sharp ZD Nelson JF Astle CM Flurkey K Rapamycin fed late in life extends lifespan in genetically heterogeneous mice. Nature. (2009) 460(7253):392–5. 10.1038/nature08221 19587680 PMC2786175

[33] Wang R Yu Z Sunchu B Shoaf J Dang I Zhao S Rapamycin inhibits the secretory phenotype of senescent cells by a Nrf2-independent mechanism. Aging Cell. (2017) 16(3):564–74. 10.1111/acel.12587 28371119 PMC5418203

[34] Das A Durrant D Koka S Salloum FN Xi L Kukreja RC. Mammalian target of rapamycin (mTOR) inhibition with rapamycin improves cardiac function in type 2 diabetic mice: potential role of attenuated oxidative stress and altered contractile protein expression. J Biol Chem. (2014) 289(7):4145–60. 10.1074/jbc.M113.521062 24371138 PMC3924280

[35] Heitman J Movva NR Hall MN. Targets for cell cycle arrest by the immunosuppressant rapamycin in yeast. Science. (1991) 253(5022):905–9. 10.1126/science.1715094 1715094

