# Supplementary material for: WGCNA and molecular docking identify hub genes for cardiac aging
Source: Front Cardiovasc Med. 2023 Apr 27;10:1146225. doi: 10.3389/fcvm.2023.1146225 (PMC10172467; doi:10.3389/fcvm.2023.1146225)
Supplement: Supplementary file 1 [file Table2.docx]

Data analyzed in this study please see:

https://www.jianguoyun.com/p/DVqt8ugQ-PSoCxim3vEEIAA
