# Supplementary material for: WGCNA and molecular docking identify hub genes for cardiac aging
Source: Front Cardiovasc Med. 2023 Apr 27;10:1146225. doi: 10.3389/fcvm.2023.1146225 (PMC10172467; doi:10.3389/fcvm.2023.1146225)
Supplement: Supplementary file 2 [file Table3.docx]

Supplementary figure legend

Figure S1 the association analysis between 10 hub genes and telomerase genes.
