# Supplementary figures and images for: WGCNA and molecular docking identify hub genes for cardiac aging
Source: Front Cardiovasc Med. 2023 Apr 27;10:1146225. doi: 10.3389/fcvm.2023.1146225 (PMC10172467; doi:10.3389/fcvm.2023.1146225)

GSE17360

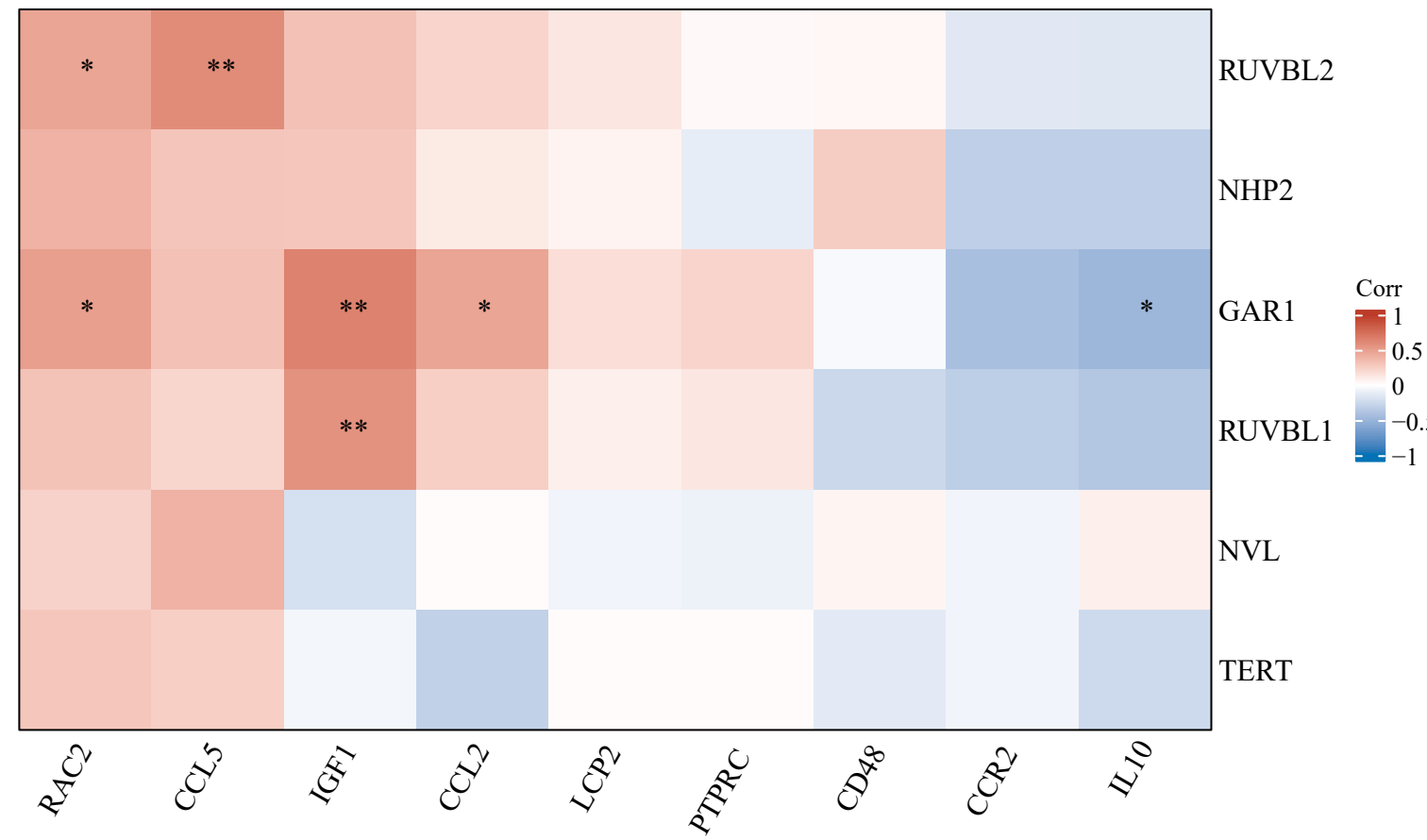

Supplement: Supplementary file 3 [file Datasheet1.pdf]
